# Bone Tunnel Enlargement after ACL Reconstruction with Hamstring Autograft Is Dependent on Original Bone Tunnel Diameter

**DOI:** 10.1055/s-0037-1603950

**Published:** 2017-06-19

**Authors:** Steffen Sauer, Martin Lind

**Affiliations:** 1 Department of Orthopaedic Surgery and Sports Medicine, Arhus University Hospital, Aarhus, Denmark

**Keywords:** ACL, tunnel widening, tunnel enlargement

## Abstract

**Background** Bone tunnel enlargement is a well-established phenomenon following anterior cruciate ligament (ACL) reconstruction, and is related to soft tissue grafts, suspension fixation devices, and absorbable implants. Severe tunnel enlargement can lead to reconstruction failure. The correlation between bone tunnel enlargement following ACL reconstruction and original bone tunnel diameter has not been elucidated.

**Purpose** To determine whether bone tunnel enlargement after ACL reconstruction with hamstring autograft is dependent on original tunnel diameter established during primary ACL reconstruction.

**Materials and Methods** A retrospective review was conducted on 56 patients scheduled for ACL revision surgery who had undergone computed tomography (CT) scanning as part of their preoperative evaluation. All patients had undergone previous hamstring ACL reconstruction. Original femoral and tibial bone tunnel diameters were extracted from operative reports, and femoral and tibial bone tunnel enlargement was assessed on CT serial sections. The correlation between original tunnel diameter and bone tunnel enlargement was investigated using regression analysis.

**Results** Mean tibial bone tunnel enlargement was significantly and inversely dependent on the original tibial bone tunnel diameter with a correlation coefficient of −0.55 per unit (7 mm = +1.93 mm, 8 mm = +1.43 mm, 9 mm = 0.83 mm, *p* = 0.007). Thus, every additional increase (mm) in diameter of the original tibial bone tunnel reduces the extend of tunnel widening by 0.55 mm.

**Conclusions** The results of this study indicate that tibial bone tunnel enlargement following ACL reconstruction is dependent on original tibial bone tunnel diameter with smaller diameter tunnels developing more tunnel enlargement than larger tunnels. The contributing factors remain unclear and need to be further investigated.

Bone tunnel enlargement is a well-established phenomenon occurring predominantly within the first 3 months following anterior cruciate ligament (ACL) reconstruction surgery. 1 2 The highest percentage of change in femoral and tibial tunnel size occurs within the first 6 weeks after surgery. 1 However, tunnel enlargement has been reported up to 2 years postoperatively. 1 3 4 The incidence of tunnel enlargement is particularly related to hamstring autografts with large reported variability ranging from 25 to 100% in femoral tunnels and 29 to 100% in tibial tunnels. 5 6 7 8 First attributed to allografts 3 9 and bone-tendon-bone (BTB) grafts, 10 11 tunnel enlargement related to hamstring autografts was first described by ĹInsalata and Harner in the late nineties. 2 Clatworthy et al proposed a multifactorial etiology of tunnel enlargement with a biochemical component after performing suspensory fixation in both hamstring and BTB grafts, finding a higher incidence of tunnel enlargement in hamstring grafts. 12 Faunoe and Kaalund reported more distinct tunnel enlargement in cortical fixation compared with transverse pin fixation of hamstring grafts, concluding that the graft fixation site in relation to the joint is crucial in the development of tunnel enlargement. 13 The exact etiology of tunnel enlargement however remains unclear and is believed to be a multifactorial process including both mechanical and biological factors. 10 12 14 15 16 17 18 19 Mechanical factors include graft tunnel-motion, especially in tunnel malposition; drill-related bone necrosis; and aggressive rehabilitation. 7 9 10 20 21 22 23 24 Biochemical factors include synovial fluid propagation and cytokine-induced osteolysis, eventually aggravated by absorbable fixation implants. 3 9 10 23 The clinical relevance of tunnel enlargement is uncertain. Although the majority of studies did not reveal a correlation between tunnel enlargement and clinical outcome, 2 3 8 9 12 17 20 23 25 26 27 some studies have recognized tunnel enlargement to be an early sign of graft failure. 28 However, a clinically *important* issue is that revision surgery is complicated by severe tunnel enlargement, eventually making the two-stage ACL revision surgery necessary. 29 Previous studies have *focused* on the correlation between bone tunnel enlargement and surgical technique, graft choice, and rehabilitation. 2 8 10 17 To our knowledge, the correlation between bone tunnel enlargement and original bone tunnel diameter has not been elucidated.

## Purpose and Hypothesis

The purpose of this study was to determine whether bone tunnel enlargement after ACL reconstruction with hamstring autograft measured on computed tomography (CT) is dependent on original tunnel diameter established during primary ACL reconstruction surgery. As both mechanical and biological causes of tunnel enlargement may theoretically be dependent on graft-tunnel contact area and bone–tendon interface, we hypothesized that smaller diameter tunnels are more susceptible to tunnel enlargement than larger tunnels.

## Methods

### Patients

All patients with accessible CT scanning of femoral and tibial bone tunnels after ACL reconstruction were identified. As CT is used as a part of preoperative revision evaluation, a cohort of 122 consecutive patients, who were scheduled for ACL revision surgery at the Aarhus University Hospital between 2013 and 2016, was identified. Of these patients, the study included 56 patients with primary ACL reconstruction using hamstring autograft and accessible primary operative reports and a new CT scan of femoral and tibial bone tunnels as part of the preoperative ACL revision evaluation. These inclusion criteria enabled CT-based evaluation of tunnel enlargement after hamstring autograft ACL reconstruction. Medical records including operative reports were assessed to identify original femoral and tibial bone tunnel diameter established during primary ACL reconstruction (range: 6–9 mm). Furthermore, patient demographics and graft fixation methods were recorded ( Table 1 ).

**Table 1:** Graft fixation methods in relation to original bone tunnel diameter established during primary ACL reconstruction (range: 6–9 mm)

Original bone tunnel diameter (mm)	6	6.5	7	7.5	8	8.5	9
Number of patients ( *n* )	1	2	15	3	16	1	17
Primary femoral ACL graft fixation							
Cortical suspension	1	2	13	3	14	1	14
Transverse pin fixation	0	0	2	0	2	0	2
Interference screw	0	0	0	0	0	0	1
Primary tibial ACL graft fixation							
Nonabsorbable screw	1	2	15	3	5	1	9
Absorbable screw	0	0	0	0	3	0	3
Nonspecified screw	0	0	0	0	8	0	5

Abbreviation: ACL, anterior cruciate ligament.

### CT Assessment

**Fig. 1:**
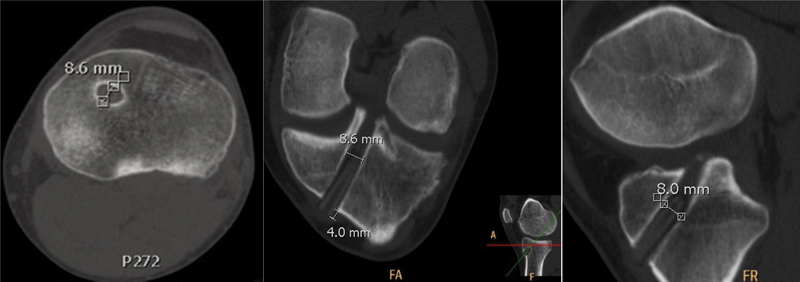
Two-dimensional (2D) computed tomography (CT) measuring method. 30 Bone tunnels are assessed in coronal, sagittal, and axial CT image planes.

Femoral and tibial bone tunnel enlargement was assessed by CT scanning (mean time from ACL reconstruction to CT tunnel measurement = 40.8 months) using the traditional two-dimensional (2D) CT method. 30 The transosseus diameter of femoral and tibial tunnels was measured at each tunnel midpoint in coronal, sagittal, and axial CT image planes using a linear measuring tool ( Fig. 1 ).

### Statistics

Mean tunnel diameter values were calculated and analysis of the correlation between original tunnel diameter and bone tunnel enlargement was investigated using regression analysis.

## Results

**Fig. 2:**
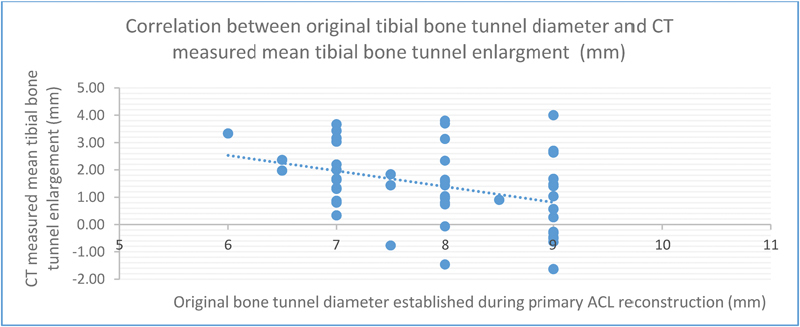
Regression analysis of the correlation between mean tibial bone tunnel enlargement and original tibial bone tunnel diameter (coefficient correlation: −0.55; confidence interval [CI]: −0.944–156; *p* = 0.007).

Tunnel enlargement from the original tunnel diameter to CT measured, follow-up diameter for both femoral and tibial bone tunnels is presented in Table 2 . For femoral tunnels, original 7-mm bone tunnels showed a mean tunnel enlargement of +0.15 mm ( *p* = 0.576). Original 8-mm bone tunnels showed a mean tunnel enlargement of −0.003 mm ( *p* = 0.987), while 9-mm original bone tunnels showed a mean tunnel enlargement of −0.16 mm ( *p* = 0.574). For tibial tunnels, original 7-mm tibial bone tunnels showed a mean tunnel enlargement of +1.93 mm ( *p* = 0.0001). Original 8-mm bone tunnels showed a mean tunnel enlargement of +1.38 mm ( *p* = 0.0001), while original 9-mm bone tunnels showed a mean tunnel enlargement of +0.83 mm ( *p* = 0.002). As seen in Fig. 2 , mean tibial bone tunnel enlargement is significantly and inversely dependent on the original tibial bone tunnel diameter with a correlation coefficient of −0.55 ( *p* = 0.007). Thus, every additional increase (mm) in diameter regarding the original tibial bone tunnel reduces the extend of tibial tunnel widening by 0.55 mm. There was no significant correlation between tunnel enlargement and the elapsed time from primary ACL reconstruction to CT follow-up measurement (mean = 40.8 months; range = 7–139 months; femoral tunnels, *p* = 0.2; tibial tunnels, *p* = 0.06). There was no significant correlation between tunnel enlargement and patient age.

**Table 2:** Tunnel enlargement presented as change in original bone tunnel diameter

Original tunnel diameter	Femoral mean bone tunnel enlargement (CI, *p* -value)	Tibial mean bone tunnel enlargement (CI, *p* -value)
7 mm	+0.15 mm; (CI: −0.4–0.7, *p* = 0.576)	+1.93 mm (CI: 1.4–2.4, *p* = 0.0001)
8 mm	−0.003 mm (CI: −0.42–0.3, *p* = 0.987)	+1.38 mm (CI: 1.1–1.7, *p* = 0.0001)
9 mm	−0.16 mm (CI: −0.7–0.4, *p* = 0.574)	+0.83 mm (CI: 0.3–1.3, *p* = 0.002)

Abbreviation: CI, confidence interval.

## Discussion

The primary finding of the this study was that tunnel enlargement of tibial bone tunnels after hamstring ACL reconstruction was inversely correlated to the original tunnel diameter, with small diameter tunnels showing more excessive tunnel enlargement than larger tunnels. Second, femoral bone tunnels did not demonstrate any significant tunnel enlargement. The dependency of bone tunnel enlargement on original bone tunnel diameter has not been described before. Clatworthy et al proposed a multifactorial etiology of tunnel enlargement with a biochemical component after performing suspensory fixation in both hamstring and BTB grafts and finding a higher incidence of tunnel enlargement in hamstring grafts. 12 Interestingly, the authors mentioned an evident difference in graft size distribution with hamstrings ranging from 6 to 10 mm and BTB grafts ranging from 9 to 13 mm. Considering the results of this study, it seems possible that the differences in original tunnel diameter *might* have contributed to the finding of more tunnel enlargement for hamstring grafts in the study of Clatworthy et al, as hamstring grafts classically have the smallest original tunnel diameters in comparison to grafts with bone blocks. The fact that femoral tunnels in comparison to tibial tunnels do not show significant tunnel enlargement has been reported before. 12 A possible explanation could be drill-related bone necrosis, which theoretically is less marked at the femoral site, as the femoral bone-reamer contact area is irrigated with arthroscopic fluid in contrast to the tibial site. Also, the femoral condylar bone has a higher density than proximal tibial bone, which could make femoral bone more resistant to tunnel enlargement after ACL reconstruction. Furthermore, tibial tunnel enlargement may be related to the biomechanical stress caused by the interference screw, which theoretically may be more distinct in smaller bone–tendon interfaces. However, a multifactorial etiology of tunnel enlargement must be assumed. The majority of studies regarding tunnel enlargement after ACL reconstruction surgery have focused on the correlation between bone tunnel enlargement and surgical technique, graft choice, fixation method, and aggressiveness of rehabilitation. 2 8 10 17 31 32 Considerably fewer studies have focused on the potential biochemical causes of tunnel enlargement including the interaction of synovial fluid with the bone–tendon interface. 33 The graft-tunnel contact area may represent a key point in the etiology of tunnel enlargement. Small diameter tunnels with less bone–tendon interface may be more susceptible for drill-related bone necrosis and biomechanical stress, especially in tunnel malposition. In addition, inflammatory cytokines in the synovial fluid may affect the tibial bone–tendon interface more excessively than femoral tunnels, as gravity tends to direct synovial fluid into the tibial bone tunnel. This could explain why femoral tunnels did not show significant tunnel enlargement. ACL reconstruction with graft diameters less than 8 mm in diameter has been shown to be associated with higher revision rates. 29 34 Insufficient graft material has been proposed as a potential cause. The results from this study suggest an additional cause for these clinical findings, as small diameter ACL reconstructions will have a higher proportion of tunnel enlargement that could result in graft fixation failure. The small and inhomogeneous patient cohort comprising different graft fixation methods represents a limitation of this study. Furthermore, all accessible CT scans represented patients who had failure of their ACL reconstruction, and therefore, could represent a population with altered biomechanics. Studies with larger cohorts are needed to investigate the correlation between original bone tunnel diameter and bone tunnel enlargement. The results of this study present a new perspective on bone tunnel enlargement etiology, as small diameter bone tunnel diameter may represent an unrecognized factor favoring bone tunnel enlargement. However, further studies are needed to revise the findings of this study.

## Conclusion

The results of this study indicate that tibial bone tunnel enlargement following ACL reconstruction is significantly and inversely dependent on the original tibial bone tunnel diameter. Every additional increase (mm) in diameter regarding the original tibial bone tunnel reduces the extend of tibial tunnel widening with 0.55 mm. The contributing factors remain unclear and need to be further investigated.

## References

[1] Buck D C Simonian P T Larson R V Borrow J Nathanson D A Timeline of tibial tunnel expansion after single-incision hamstring anterior cruciate ligament reconstruction Arthroscopy 2004 20 01 34 36 14716276 10.1016/j.arthro.2003.10.011

[2] L'Insalata J C Klatt B Fu F H Harner C D Tunnel expansion following anterior cruciate ligament reconstruction: a comparison of hamstring and patellar tendon autografts Knee Surg Sports Traumatol Arthrosc 1997 5 04 234 238 9430573 10.1007/s001670050056

[3] Jackson D W Windler G E Simon T M Intraarticular reaction associated with the use of freeze-dried, ethylene oxide-sterilized bone-patella tendon-bone allografts in the reconstruction of the anterior cruciate ligament Am J Sports Med 1990 18 01 1 10; discussion 10–11 10.1177/036354659001800101 2301680

[4] Sgaglione N A Douglas J A Allograft bone augmentation in anterior cruciate ligament reconstruction Arthroscopy 2004 20 02 171 177 15243454 10.1016/j.arthro.2004.04.030

[5] Fules P J Madhav R T Goddard R K Newman-Sanders A Mowbray M A Evaluation of tibial bone tunnel enlargement using MRI scan cross-sectional area measurement after autologous hamstring tendon ACL replacement Knee 2003 10 01 87 91 12649033 10.1016/s0968-0160(02)00086-8

[6] Jansson K A Harilainen A Sandelin J Karjalainen P T Aronen H J Tallroth K Bone tunnel enlargement after anterior cruciate ligament reconstruction with the hamstring autograft and endobutton fixation technique. A clinical, radiographic and magnetic resonance imaging study with 2 years follow-up Knee Surg Sports Traumatol Arthrosc 1999 7 05 290 295 10525698 10.1007/s001670050166

[7] Nebelung W Becker R Merkel M Röpke M Bone tunnel enlargement after anterior cruciate ligament reconstruction with semitendinosus tendon using Endobutton fixation on the femoral side Arthroscopy 1998 14 08 810 815 9848590 10.1016/s0749-8063(98)70015-5

[8] Segawa H Omori G Tomita S Koga Y Bone tunnel enlargement after anterior cruciate ligament reconstruction using hamstring tendons Knee Surg Sports Traumatol Arthrosc 2001 9 04 206 210 11522075 10.1007/s001670100201

[9] Roberts T S Drez D Jr McCarthy W Paine R Anterior cruciate ligament reconstruction using freeze-dried, ethylene oxide-sterilized, bone-patellar tendon-bone allografts. Two year results in thirty-six patients Am J Sports Med 1991 19 01 35 41 2008928 10.1177/036354659101900106

[10] Fahey M Indelicato P A Bone tunnel enlargement after anterior cruciate ligament replacement Am J Sports Med 1994 22 03 410 414 8037283 10.1177/036354659402200318

[11] Peyrache M D Djian P Christel P Witvoet J Tibial tunnel enlargement after anterior cruciate ligament reconstruction by autogenous bone-patellar tendon-bone graft Knee Surg Sports Traumatol Arthrosc 1996 4 01 2 8 8819056 10.1007/BF01565989

[12] Clatworthy M G Annear P Bulow J U Bartlett R J Tunnel widening in anterior cruciate ligament reconstruction: a prospective evaluation of hamstring and patella tendon grafts Knee Surg Sports Traumatol Arthrosc 1999 7 03 138 145 10401649 10.1007/s001670050138

[13] Fauno P Kaalund S Tunnel widening after hamstring anterior cruciate ligament reconstruction is influenced by the type of graft fixation used: a prospective randomized study Arthroscopy 2005 21 11 1337 1341 16325084 10.1016/j.arthro.2005.08.023

[14] Clatworthy M G Bartelett J Howell S The effect of graft fixation techniques on tunnel widening in hamstring ACL reconstruction Arthroscopy 1999 15(Suppl):5

[15] Harris N L Indelicato P A Bloomberg M S Meister K Wheeler D L Radiographic and histologic analysis of the tibial tunnel after allograft anterior cruciate ligament reconstruction in goats Am J Sports Med 2002 30 03 368 373 12016077 10.1177/03635465020300031101

[16] Hersekli M A Akpinar S Ozalay M Tunnel enlargement after arthroscopic anterior cruciate ligament reconstruction: comparison of bone-patellar tendon-bone and hamstring autografts Adv Ther 2004 21 02 123 131 15310085 10.1007/BF02850339

[17] Höher J Möller H D Fu F H Bone tunnel enlargement after anterior cruciate ligament reconstruction: fact or fiction? Knee Surg Sports Traumatol Arthrosc 1998 6 04 231 240 9826805 10.1007/s001670050105

[18] Wilson T C Kantaras A Atay A Johnson D L Tunnel enlargement after anterior cruciate ligament surgery Am J Sports Med 2004 32 02 543 549 14977688 10.1177/0363546504263151

[19] Wright R W Dunn W R Amendola A Risk of tearing the intact anterior cruciate ligament in the contralateral knee and rupturing the anterior cruciate ligament graft during the first 2 years after anterior cruciate ligament reconstruction Am J Sports Med 2007 35 07 1131 1134 17452511 10.1177/0363546507301318

[20] Linn R M Fischer D A Smith J P Burstein D B Quick D C Achilles tendon allograft reconstruction of the anterior cruciate ligament-deficient knee Am J Sports Med 1993 21 06 825 831 8291633 10.1177/036354659302100611

[21] Otsuka H Ishibashi Y Tsuda E Sasaki K Toh S Comparison of three techniques of anterior cruciate ligament reconstruction with bone-patellar tendon-bone graft. Differences in anterior tibial translation and tunnel enlargement with each technique Am J Sports Med 2003 31 02 282 288 12642266 10.1177/03635465030310022101

[22] Sakai H Yajima H Hiraoka H The influence of tibial fixation on tunnel enlargement after hamstring tendon anterior cruciate ligament reconstruction Knee Surg Sports Traumatol Arthrosc 2004 12 05 364 370 14661074 10.1007/s00167-003-0455-7

[23] Schulte K Majewski M Irrgang J J Radiographic tunnel changes following arthroscopical reconstruction: autograft versus allograft Arthroscopy 1995 11 372 373

[24] Simonian P T Erickson M S Larson R V O'kane J W Tunnel expansion after hamstring anterior cruciate ligament reconstruction with 1-incision EndoButton femoral fixation Arthroscopy 2000 16 07 707 714 11027754 10.1053/jars.2000.4635

[25] Fink C Zapp M Benedetto K P Hackl W Hoser C Rieger M Tibial tunnel enlargement following anterior cruciate ligament reconstruction with patellar tendon autograft Arthroscopy 2001 17 02 138 143 11172242 10.1053/jars.2001.21509

[26] Lind M Feller J Webster K E Bone tunnel widening after anterior cruciate ligament reconstruction using EndoButton or EndoButton continuous loop Arthroscopy 2009 25 11 1275 1280 19896050 10.1016/j.arthro.2009.06.003

[27] Webster K E Feller J A Hameister K A Bone tunnel enlargement following anterior cruciate ligament reconstruction: a randomised comparison of hamstring and patellar tendon grafts with 2-year follow-up Knee Surg Sports Traumatol Arthrosc 2001 9 02 86 91 11354858 10.1007/s001670100191

[28] Siebold R Kiss Z S Morris H G Effect of compaction drilling during ACL reconstruction with hamstrings on postoperative tunnel widening Arch Orthop Trauma Surg 2008 128 05 461 468 17899135 10.1007/s00402-007-0443-3

[29] Rizer M Foremny G B Rush A III Anterior cruciate ligament reconstruction tunnel size: causes of tunnel enlargement and implications for single versus two-stage revision reconstruction Skeletal Radiol 2017 46 02 161 169 27885380 10.1007/s00256-016-2535-z

[30] Crespo B Aga C Wilson K J Measurements of bone tunnel size in anterior cruciate ligament reconstruction: 2D versus 3D computed tomography model J Exp Orthop 2014 1 01 2 26914747 10.1186/s40634-014-0002-0 PMC4648836

[31] Hantes M E Mastrokalos D S Yu J Paessler H H The effect of early motion on tibial tunnel widening after anterior cruciate ligament replacement using hamstring tendon grafts Arthroscopy 2004 20 06 572 580 15241306 10.1016/j.arthro.2004.04.069

[32] Iorio R Vadalà A Argento G Sanzo V D Ferretti A Bone tunnel enlargement after ACL reconstruction using autologous hamstring tendons: a CT study Int Orthop 2007 31 01 49 55 16683112 10.1007/s00264-006-0118-7 PMC2267545

[33] Zysk S P Fraunberger P Veihelmann A Tunnel enlargement and changes in synovial fluid cytokine profile following anterior cruciate ligament reconstruction with patellar tendon and hamstring tendon autografts Knee Surg Sports Traumatol Arthrosc 2004 12 02 98 103 14504722 10.1007/s00167-003-0426-z

[34] Spragg L Chen J Mirzayan R Love R Maletis G The effect of autologous hamstring graft diameter on the likelihood for revision of anterior cruciate ligament reconstruction Am J Sports Med 2016 44 06 1475 1481 27002103 10.1177/0363546516634011

